# This story shall the good [surgeon] teach

**DOI:** 10.1308/rcsann.2026.0074

**Published:** 2026-07-01

**Authors:** B Rogers

**Affiliations:** *Annals* Editor-in-Chief

**Figure:**
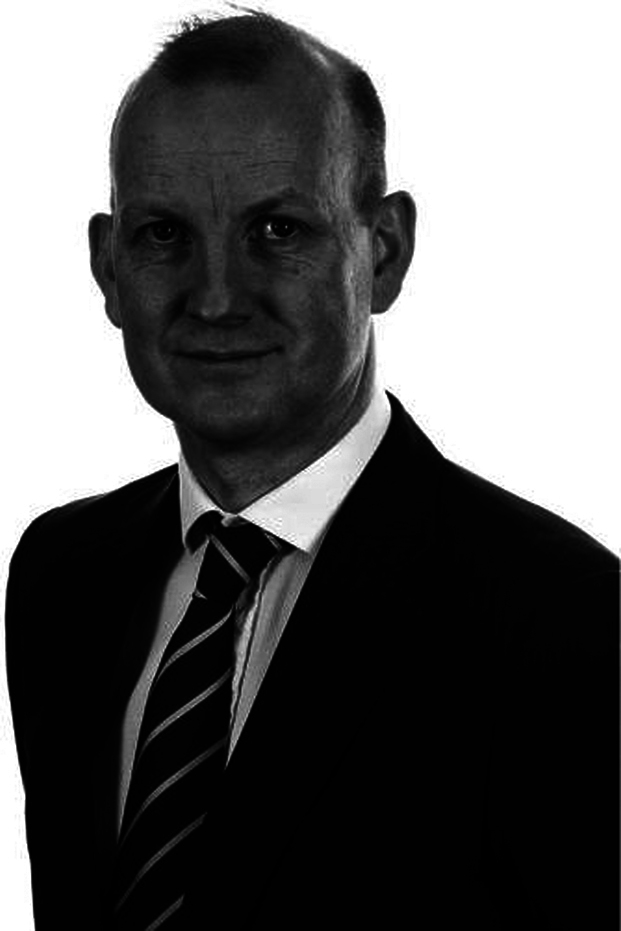


The July 2026 *Annals of the Royal College of Surgeons of England* is a themed issue focusing on frailty, perhaps one of the most critical determinants in surgical outcomes. Given the ageing population of Western society (the proportion of people living over 85 years in the UK is set to double in the next 20 years),^1^ it must attract a greater focus of clinical surgical research. As someone who obtained the Diploma in Geriatric Medicine and as it is also a topic relevant to the daily practice of many surgeons, I am delighted to highlight two invited guest editorials from leading experts in this field.

In ‘Frailty: new horizons in older patients needing surgery’, Dr Iain Wilkinson provides a superb overview of the ideas and concepts in frailty medicine. How we identify frailty, its management, decision making and the future of frailty care are all considered.

In ‘Frailty in emergency surgery: expanding the role of biomarkers’, Professor Susan Moug and colleagues assess the condition as a dynamic physiological state that affords evaluation with biomarkers. Understanding frailty as a critical determinant of surgical outcomes will enable surgical teams to develop targeted interventions to improve patient recovery.

Facilitating excellent surgical care (particularly for vulnerable patients such as frail individuals) is the *raison d’*ê*tre* of the Royal College of Surgeons of England (RCS England).^2^ Achieving excellent surgical care is undoubtedly multifaceted. Research, optimising patient outcomes and setting professional standards are the key tenets of RCS England. Since 1947, the *Annals* has continued to play a key role in all these facets of our profession. More recently, last month The *Annals* received its highest ever impact factor (2.0) and is now ranked 137 out of 321 surgical journals worldwide. This excellent achievement is testament to great research studies, thorough peer review and a dedicated publication team.

The future of surgery is often described in terms of technology – robotics, artificial intelligence, augmented reality and precision medicine. Indeed, RCS England annually hosts the inspiring Future of Surgery Festival.^3^ However, amid the dazzling promise of machines and algorithms, one human quality remains indispensable: passion. Without it, innovation risks becoming sterile; with it, the evolution of surgery can remain both transformative and deeply human.

Passion has always been the quiet force behind surgical progress. It is what drove early pioneers to operate beyond the limits of known science, and it continues to propel modern surgeons to refine techniques, challenge dogma and improve patient outcomes.

In his *Nicomachean Ethics*, Aristotle proposed that we should all aim for *eudaimonia* (to flourish, and have passion for work, knowledge, research and reasoning). Surgeons should strive for our profession to be structured to maximise this.

Looking ahead, it is tempting to focus exclusively on what technology can achieve. Nevertheless, the true measure of progress in surgery will not be defined solely by faster procedures or smaller incisions. It will be defined by how well these advances serve patients – and that depends on the people who wield them. Passion ensures that surgery remains not just a science but an art.

There is some irony that as metrics are increasingly valued and utilised in surgery, passion for our profession (perhaps the most important quality of a surgeon) affords no metric. If a good surgical career were considered a story, Shakespeare would suggest: “This story shall the good [surgeon] teach” (*Henry V*).

This editorial marks the end of my six-year tenure as editor-in-chief of the *Annals*. It has been an immense pleasure and honour to hold this role but it would have been impossible without the dedication and hard work of the editorial board, reviewers and submitting authors. I would like especially to express my sincere thanks to the publishing team at RCS England; fellows and members should be grateful for all the work you do. I wish the incoming editor-in-chief, Professor Kamal Mahawar, the very best – the journal is in safe hands.

RCS England continues to thrive only if people have passion. I am confident that the *Annals* will flourish and progress.
